# Using Dynamic Causal Bayesian Networks to Assess the Role of Patient-Centered Care and Individual-Level Barriers on Viral Suppression Changes Among a Cohort of People with HIV

**DOI:** 10.1007/s10461-026-05082-w

**Published:** 2026-02-22

**Authors:** Mary Jo Trepka, Samantha Gonzales, Melissa K. Ward, Kristopher P. Fennie, Diana M. Sheehan, Michele Jean-Gilles, Jessy Devieux, Aaliyah Gray, Robert Ladner, Changwon Yoo

**Affiliations:** 1 Department of Epidemiology, Robert Stempel College of Public Health and Social Work, Florida International University, 11200 SW 8th St, Academic Health Center 5, Miami, FL 33199 USA; 2 Department of Biostatistics, Robert Stempel College of Public Health and Social Work, Florida International University, 11200 SW 8th St, Academic Health Center 5, Miami, FL 33199 USA; 3 Division of Natural Sciences, New College of Florida, 5800 Bay Shore Road, Sarasota, FL 34243 USA; 4 Department of Health Promotion and Disease Prevention, Robert Stempel College of Public Health and Social Work, Florida International University, 11200 SW 8th St, Academic Health Center 5, Miami, FL 33199 USA; 5 Behavioral Science Research Corp, 2121 Ponce de Leon Boulevard, Suite 240, Coral Gables, FL 33134 USA

**Keywords:** HIV, HIV viral suppression, Patient, Centered care, Patient, Provider engagement, Physician, Patient relations, Dynamic causal Bayesian network analyses

## Abstract

To assess the impact of changes in barriers to HIV viral suppression and the moderating role of patient-provider relationships, we conducted a retrospective cohort study using dynamic causal Bayesian network analyses on the first two available needs assessments between May 2020 and December 2022 for clients of the Miami-Dade County Ryan White Program (RWP) Part A Program. A cohort of 1539 individuals was formed using data from the RWP medical case management dataset. These records were merged with summary patient-centered care measures of their provider that were obtained from an earlier survey of clients in the program. Of the 1539 individuals in the longitudinal cohort, 86.2% were virally suppressed at time point 1 and 87.5% at time point 2. At time point 2, 144 (9.4%) were newly suppressed, and 124 (8.1%) were newly non-suppressed. Of the modifiable factors assessed, drug use and tobacco use significantly predicted a low probability of viral load suppression, while provider rating and provider’s Health Care Relationship Trust Score significantly predicted a high probability of viral load suppression, although the associations with drug use and tobacco use were stronger than those with any provider variables. Results highlight the need for interventions promoting strong patient-provider relationships and targeting reductions in tobacco and illegal drug use with priorities based on the prevalence of tobacco and illegal drug use within specific RWP client populations.

**Supplementary Information:**

The online version contains supplementary material available at 10.1007/s10461-026-05082-w.

## Introduction

Of the 1,018,430 people ≥ 13 years of age living with diagnosed HIV in the United States, an estimated 65.1% were virally suppressed in 2022 [1], and this percentage was lower among Black/African Americans (60.5%) and Hispanic/Latinos (64.3%) than among Whites (70.8%) [1]. This is a critical problem given that Black/African Americans and Hispanic/Latinos are disproportionately represented among people with diagnosed HIV with a prevalence in 2023 of 1037.5 per 100,000 and 439.7 per 100,000, respectively compared with 157.2 per 100,000 among Whites [2]. The US Ending the HIV Epidemic target was to have 95% of people with diagnosed HIV in the United States (US) be virally suppressed by 2025 and to maintain this target through 2030 [2]. Achieving the 95% goal moving forward will require considerable improvements, particularly among people who face the most challenging barriers to antiretroviral therapy (ART) adherence and retention in care.

Viral suppression requires engagement in care and ART adherence. There is substantial evidence that low socioeconomic status (SES) and accompanying unmet needs related to housing, transportation, and food pose significant barriers to engagement in HIV care, ART adherence, and viral suppression [3–7]. Mental health problems [7–9], heavy alcohol use or abuse [10–12], illegal drug use [7, 13–19] and dependence [20], tobacco use [21–27], and lack of social support [28–30] are also well-documented barriers.

The presence and severity of any of these barriers can change over the course of a person’s life. As more people are living longer with HIV, the likelihood of changes in life circumstances increases. However, there have been few studies that have examined the effects of changes in barriers on ART adherence and viral suppression. Several studies focusing on alcohol use found that initiation or increased alcohol use was associated with a lower likelihood of ART adherence [31, 32] and viral suppression [31–33] although one found that adherence decreased when alcohol use both increased and decreased, a finding the authors suggested may be due to underlying alcohol use disorder or a proxy for unstable life circumstances [34]. New onset of substance abuse has been found to be associated with declining adherence [35, 36] and lack of viral suppression [36].

Addressing barriers to engagement in care and adherence is challenging within the health care system because many barriers are driven by societal and structural factors such as poverty and lack of affordable housing, which health care providers cannot control. However, health care providers may be able to reduce the effects of these barriers on a patient’s HIV care outcomes. Strong, trusting relationships with providers and lack of provider mistrust are associated with patients being successful in engagement in care and ART adherence [37–52]. For viral suppression, results have been mixed with some studies finding that such relationships with providers are associated with viral suppression [38, 39, 46, 53] and others not finding a relationship [45, 50, 52]. In a previous study, using a cross-sectional causal Bayesian network analysis, our team identified that Healthcare Relationship Trust Score moderated the association between low household income and lack of durable viral suppression cross-sectionally among Ryan White Program clients medically case managed during 2019 [54]. We identified no longitudinal studies that assessed a potential moderating role of a strong patient-provider relationship on the effect of adverse changes in psychosocial factors on HIV viral suppression.

To address the limited data on the effect of dynamic changes in individual factors on viral suppression (which are likely to increasingly occur as people with HIV age) as well as the role of patient-provider relationships in moderating adverse effects of changes, we conducted a retrospective cohort study. Because of the complexity involved in considering numerous factors over time, we used dynamic causal Bayesian networks to analyze the effect of a change in mental health status, social support, tobacco use, drug use, poverty status, employment status, and unmet needs on changes in viral suppression and to assess if the quality of the patient-provider relationship moderated the effect of adverse changes on viral load suppression.

## Methods

### Design and Setting

This longitudinal study was of clients ≥ 18 years of age medically case managed as of May 2020 by the Miami-Dade County Ryan White Part A Program (RWP). The RWP pays for HIV medical care, medications, medical case management, and support services for low-income individuals in several U.S. metropolitan areas, selected based on the number of AIDS cases during the last 5 years [55]. During Fiscal Year 2022, the Miami-Dade County RWP served 8599 clients, roughly 30% of people diagnosed with HIV in the county [56].

### Study Population and Administrative Data Set

Administrative files, including client profile, needs assessments, financial assessment, laboratory and billing data for the period May 2020 − December 2022 were de-identified, coded with a random study code, and transferred to researchers. The files were merged by researchers using the study code. The study population for the present analysis included clients who had at least two needs assessments and two viral load values during May 2020 − December 2022.

The selection of demographic and psychosocial characteristics for this analysis was guided by an adaptation of the Andersen Behavioral Model for Health Services Utilization [57] specifically for HIV care [58, 59]. The adapted model posits that HIV care-related behaviors are influenced by individual-level demographic, psychosocial, and need characteristics as well as the health care and community environment.

Race/ethnicity was classified into four groups: Non-Hispanic Black (excluding Haitian), Hispanic of any race, Haitian, and Non-Hispanic White/Other. Born in the United States meant born in one of the 50 states. Language was the client’s preferred language according to the Ryan White Program administrative database. The poverty level and employment variables came from the financial records used to ensure that participants qualify for the Ryan White Program. The other individual-level variables were answers to questions administered by medical case managers during the needs assessments that are normally every 6 months; the wording of the questions is in Table 1.

**Table 1:** Viral suppression by demographic and psychosocial factors at timepoints 1 and 2

Variable	Time point 1							Time point 2						
	Total	Suppressed		Not suppressed				Total	Suppressed		Not suppressed			
	N	n	%	n	%	Test-statistic	*P*-value^a^	N	n	%	n	%	Test-statistic	*P*-value^a^
Total	1539	1327		212				1539	1347		192			
Demographic Factors														
*Age group (years)*														
18–24	35	24	1.8	11	5.2	12.40	0.01	22	16	1.2	6	3.1	20.39	0.004
25–34	268	230	17.3	38	17.9			245	197	14.6	48	25.0		
35–49	572	500	37.7	72	34.0			573	509	37.8	64	33.3		
50–64	623	541	40.8	82	38.7			644	573	42.5	71	37.0		
≥ 65	41	32	2.4	9	4.3			55	52	3.9	3	1.6		
*Current gender*														
Male	1248	1075	81.0	173	81.6	3.29	0.19	1248	1103	81.9	145	75.5	4.73	0.094
Female	271	232	17.5	39	18.4			271	228	16.9	43	22.4		
Transgender female	20	20	1.5	0	0.0			20	16	1.2	4	2.1		
*Race/ethnicity*														
Non-Hispanic Black^b^	254	202	15.2	52	24.5	22.66	<.0001	254	196	14.6	58	30.2	39.89	<.0001
Hispanic	1045	924	69.6	121	57.1			1045	941	69.9	104	54.2		
Haitian	140	110	8.3	30	14.2			140	115	8.5	25	13.0		
Other	100	91	6.9	9	4.3			100	95	7.1	5	2.6		
*Preferred language*														
English	429	351	26.5	78	36.8	20.43	0.0001	429	348	25.8	81	42.2	31.50	<.0001
Spanish	941	838	63.2	103	48.6			941	858	63.7	83	43.2		
Creole	113	88	6.6	25	11.8			113	92	6.8	21	10.9		
Other	56	50	3.8	6	2.8			56	49	3.6	7	3.7		
*Born In US*^*c*^														
No	1184	1040	78.4	144	67.9	11.24	0.0008	1184	1069	79.4	115	59.9	35.88	<.0001
Yes	355	287	21.6	68	32.1			355	278	20.6	77	40.1		
Neighborhood of Residence at Time Point 1														
*Neighborhood SES vulnerability (higher index equals more SES vulnerable, i.e., more impoverished*^*d*^*)*														
<.64 (least vulnerable)	590	538	40.5	52	24.5	23.44	<.0001	590	545	40.5	45	23.4	22.27	<.0001
.64 −.87	475	405	30.5	70	33.0			475	408	30.3	67	34.9		
≥.88 (most vulnerable)	474	384	28.9	90	42.5			474	394	29.3	80	41.7		
Individual Psychosocial Factors Measured at Time Points 1 and 2														
*Poverty level (household income as percentage of FPL)*														
≥ 200% (least impoverished)	337	311	23.4	26	12.3	17.12	0.0002	432	391	29.0	41	21.4	14.59	0.0007
100 − 199%	592	512	38.6	80	37.7			558	499	37.1	59	30.7		
< 100% (most impoverished)	610	504	38.0	106	50.0			549	457	33.9	92	47.9		
*Employed (“Are you currently working?”)*														
Yes	986	867	65.3	119	56.1	6.72	0.0095	1026	918	68.2	108	56.3	10.71	0.001
No	553	460	34.7	93	43.9			513	429	31.9	84	43.8		
*Food insecurity (“In the past four weeks did you worry that there was not enough food for everyone in household to eat?”)*														
No	1422	1231	92.8	191	90.1	1.86	0.17	1436	1262	93.7	174	90.6	2.53	0.11
Yes	117	96	7.2	21	9.9			103	85	6.3	18	9.4		
*Housing situation (“What is your current living arrangement?”)*														
Safe permanent	1437	1248	94.1	189	89.2	19.18	0.0003	1442	1277	94.8	165	85.9		<.0001^a^
Unsafe permanent	37	34	2.6	3	1.4			37	30	2.2	7	3.7		
In institution	15	12	0.9	3	1.4			11	8	0.6	3	1.6		
Non-permanent (homeless, transient, or transitional)	50	33	2.5	17	8.0			49	32	2.4	17	8.9		
*Ability to meet expenses (“How are you doing meeting your monthly expenses?”)*														
Meets all monthly expenses	966	832	62.7	134	63.2	24.95	<.0001	887	788	58.5	99	51.6	11.85	0.01
Meets most monthly expenses	359	330	24.9	29	13.7			405	359	26.7	46	24.0		
Meets some monthly expenses	135	105	7.9	30	14.2			162	131	9.7	31	16.2		
Rarely meets monthly expenses	79	60	4.5	19	9.0			85	69	5.1	16	8.3		
*Medical transportation access (“Do you have access to transportation for medical case management, healthcare, dental and other appointments?”)*														
Yes	1358	1171	88.2	187	88.2	0.0002	1.0	1353	1187	88.1	166	86.5	0.44	0.51
No	181	156	11.8	25	11.8			186	160	11.9	26	13.5		
*Social support system (“Do you have a social support system you can depend on?”)*														
Has	1214	1056	79.6	158	74.5	2.80	0.094	1244	1086	80.6	158	82.3	0.30	0.58
Does not have	325	271	20.4	54	25.5			295	261	19.4	34	17.7		
*Emotional feelings (“How are you feeling emotionally these days?”)*														
Excellent	256	229	17.3	27	12.7	22.59	<.0001	249	225	16.7	24	12.5	30.62	<.0001
Very good	487	434	32.7	53	25.0			500	446	33.1	54	28.1		
Good	694	590	44.5	104	49.1			689	605	44.9	84	43.8		
Fair or Poor	102	74	5.6	28	13.2			101	71	5.3	30	15.6		
*Any drug use (Says “yes” to either currently using injection drugs or currently using non-injection drugs)*														
No	1474	1278	96.3	196	92.5	6.71	0.096	1455	1287	95.6	168	87.5	21.08	< 0.0001
Yes	65	49	3.7	16	7.6			84	60	4.5	24	12.5		
*Tobacco use (“Do you use tobacco products?”)*														
No	1420	1236	93.1	184	86.8	10.33	0.001	1410	1253	93.0	157	81.8	27.70	<.0001
Yes	119	91	6.9	28	13.2			129	94	7.0	35	18.2		

*FPL* Federal Poverty Level. Please note that totals of column percentages may slightly exceed 100% due to rounding

^a^Chi-square was the test statistic in all cases except for housing status at timepoint 2 with viral load suppression at timepoint 2, in which case Fishers Exact test was used due to expected frequency of more than 25% of cells < 5

^b^Non-Hispanic Black excludes people who identified as having Haitian ethnicity

^c^Those born in Puerto Rico or other US territories are classified as non-US born

^d^Neighborhood SES: Neighborhood socioeconomic status as measured by ZIP Code socioeconomic social vulnerability index. Higher level means more vulnerability (i.e. lower SES status). Cut-offs for tertile groups were based on tertiles of Florida ZIP Code tabulation areas

Neighborhood environment was measured using the SES theme of the Social Vulnerability Index (SVI) calculated at the ZIP Code tabulation area (ZCTA) level using data from the American Community Survey 2014–2019 5-year population estimates for ZCTAs [60] and methods developed by the Centers for Disease Control and Prevention [61]. The socioeconomic SVI is the total of the ranking of percentage of people below 150% of Federal Poverty Level (FPL), unemployment rate, per capita income (reverse coded), and percentage of people 25 and older with no high school diploma. Higher scores on the index, which ranges from 0 to 1, indicate greater SES vulnerability (i.e., low SES). Tertiles of Florida ZCTAs were used to classify areas into high, middle, and low SES vulnerability, with low SES SVI (index < 0.64) indicating wealthiest ZCTAs and high (index ≥ 0.88) indicating poorest ZCTAs. The ZCTA-level socioeconomic SVI tertile, hereafter referred to as *Neighborhood SES Vulnerability*, was matched with each client record using the client’s residential ZIP Code at the first health assessment.

Viral load data are obtained from RWP-affiliated providers and entered into the RWP administrative database on an ongoing basis. Viral load suppression was defined as having a viral load test result < 200 copies per ml.

### Measuring Patient-Centered Care and Health Care Environment

The medical case management (MCM) agency size (i.e., volume of clients served by agency) and provider patient volume (number of RWP clients served by each medical provider) were determined from the administrative dataset and were classified as < 500 vs. ≥ 500 clients for MCM agency based on a bimodal distribution in number of RWP clients served. In 2021, the eight agencies classified as small had 20–220 clients, and the five classified as large had 712–1715 clients. The medical providers were classified into three groups based on tertiles of the number of RWP clients each provider served: < 100, 100–199, and ≥ 200 RWP clients. MCM agencies were also categorized based on whether mental health treatment or substance abuse treatment were provided. The MCM agency variables and provider volume were merged with the individual-level administrative data based on the specific MCM agency and provider of the client at time point 1 (T1) and time point 2 (T2).

We obtained patient-centered care (PCC) practice data from completed interviews of 1352 RWP clients of the 50 HIV medical care providers with the largest number of RWP clients in the system from October 2020 to March 2022. The details of the survey and its measurements are described elsewhere [54].

After the survey was completed, we used multiple imputation using naïve Bayesian classifier to deal with missingness. We calculated six measures of PCC from items in the survey (Supplementary Table 1): 1) the Health Care Relationship (HCR) Trust Scale score calculated from 13-items about how frequently provider treats the patient respectfully, as an individual person, and as a partner in care as well as the quality of the communication [62]; 2) a global rating (0 − 10) of provider from the Agency for Healthcare Research and Quality Consumer Assessment of Healthcare Providers and Systems (CAHPS 3.0) [63]; 3) an item about provider or provider’s staff asking if there are things that make it hard for patient to take care of their health (CAHPS 3.0) [63]); 4) an item about if provider or provider’s staff ask patient about things in their life that worry them or cause them stress (CAHPS 3.0) [63]; 5) a summary measure about quality of provider communication from four CAHPS 3.0 questions [63]; and 6) an item about how strongly patients agree that their provider knows them as a person [38]. The 13 HCR Trust Scale items were totaled and ranged from 0 (worst) to 52 (best) score [54], and the provider communication measure was obtained after summing 4 questions (Supplementary Table 1). Means of the HCR Trust Scale score and the other 5 measures were calculated for each provider.

To apply a closed-form causal Bayesian structure scoring matrix with Dirichlet priors, the PCC variables were categorized into three levels based on the distribution of values for the providers: low (values < 1 standard deviation [SD] below the mean), medium (values ≥ 1 SD below the mean and < 1 SD above the mean), and high (values ≥ 1 SD above the mean) except for provider knows as person, which was categorized into two levels: low (values < 1 SD above the mean) and high (values ≥ 1 SD above the mean). The six three-level PCC variables were named *Provider HCR Score, Provider Rating, Provider Asks About Challenges, Provider Asks About Worries, Provider Communication,* and *Provider Knows As Person.* PCC variables were merged with all RWP eligible clients in the administrative data based on the HIV provider at T1 and T2.

### Statistical Analysis

#### Dynamic Causal Bayesian Network Background

A Bayesian network is a probabilistic graphical model that represents a multivariate joint probability distribution through a directed acyclic graph. Each node denotes a random variable, while directed edges signify conditional dependencies. By encoding the local Markov property, Bayesian networks facilitate efficient probabilistic inference and causal reasoning, enabling the calculation of posterior distributions for latent variables upon observing new evidence [64]. Causal Bayesian networks (CBNs) are a subset of Bayesian networks where the directed edges (arcs) can be interpreted as causal relationships rather than mere statistical correlations [65]. Similarly, dynamic causal Bayesian networks (DCBNs) extend this framework into the temporal domain. As a form of dynamic Bayesian network that explicitly models time, DCBNs allow for the analysis of longitudinal data, with their arcs representing causal influences across different time steps [66].

#### Dynamic Causal Bayesian Network Development

After examining the association between all variables and viral suppression using chi-square tests, dynamic causal Bayesian networks (DCBN) were used to examine the relationships between demographic, psychosocial, neighborhood SES vulnerability, medical case management environment, and provider PCC variables on viral suppression at the first 2 time points that were available for each client. To qualify as a time point, the health assessments had to be at least 5 months apart and the viral loads at least 2 months apart with the viral load having to be collected within 3 months before or after the health assessment.

**Fig. 1:**
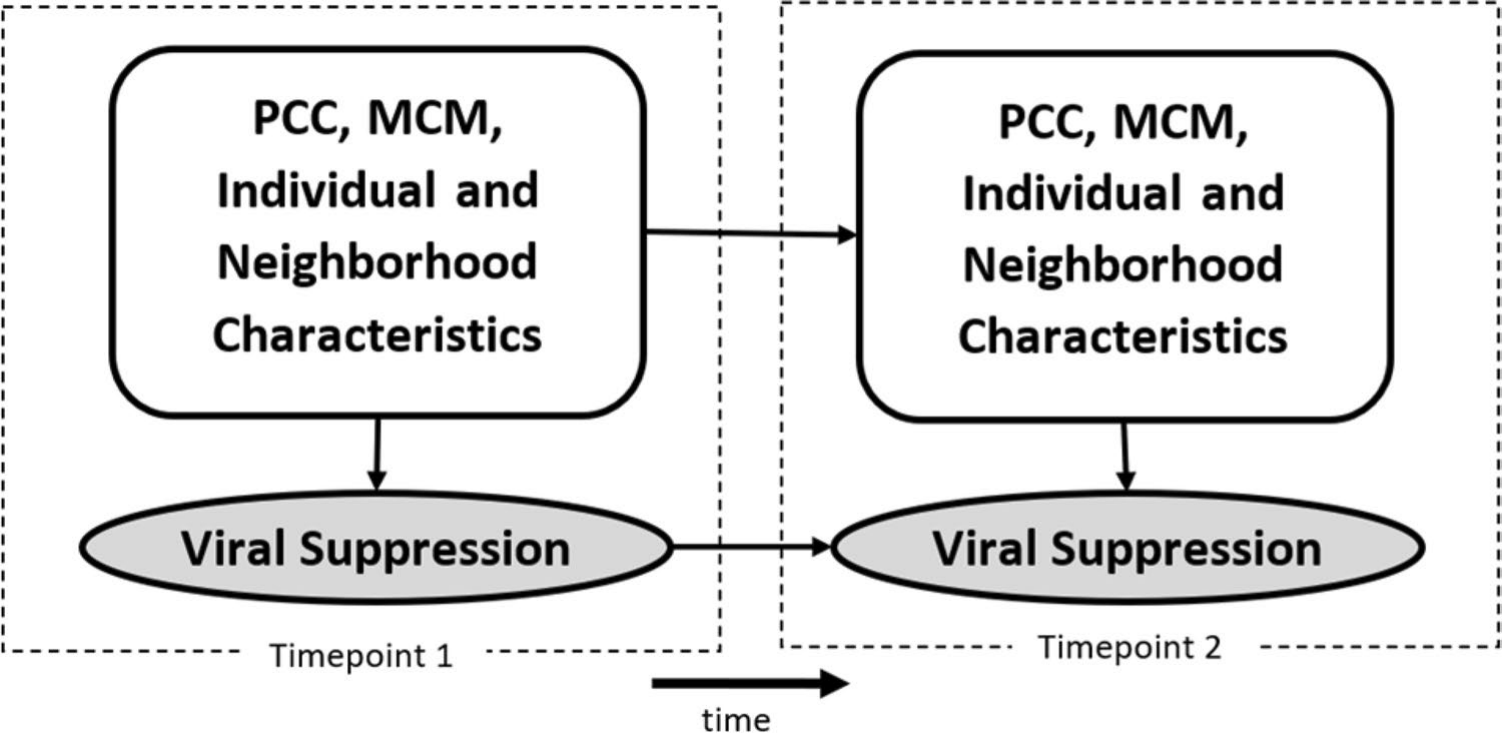
Dynamic Causal Bayesian Network Model (Each rectangle represents 4 subnetworks: patient centered care (PCC), medical case management (MCM) agency, individual patient, and neighborhood characteristics). Figure created in Microsoft Word

Figure 1 illustrates a DCBN that represents a plausible temporal causal relationship between measured variables, where time is modeled as being discrete. Each time point is associated with a causal Bayesian network that represents the state of the model variables at that time point.

Using the methods of causal discovery [67–74], we identified the best fitting network among all the modeled variables for each subnetwork shown in Fig. 1 (i.e. the PCC, MCM agency, patient and neighborhood characteristics). Furthermore, we searched for a maximum a posteriori subnetwork by combining the prior and the likelihood based on the collected data of the subnetwork.

We utilized Banjo version 2.2.0 [75] to learn the static Bayesian networks at T1 and T2 from the data. For each time point, we conducted three independent network analyses for a sequence of running times (1 h, 4 h, 16 h, and 32 h) and scored each structure using Bayesian Dirichlet equivalence [76]. In each subsequent set of searches, one search utilized the best scoring structure from the previous set, while the other two searches used random structures. The search was stopped when the current best network’s score was less than 0.001% better than the previous best network’s score.

After determining the static T1 and T2 structures, we ran dynamic causal Bayesian analyses using a similar strategy to the static analysis, which resulted in a 1 h, 4 h, and 8 h search. We searched for the optimal DCBN by combining the T1 and T2 static structures, using this combined structure as the initial dynamic network for our searches. To ensure temporal consistency, we mandated arcs from each variable at T1 to its corresponding variable at T2 (e.g., an arc from *Viral Suppression 1* to *Viral Suppression 2*). During the optimal DCBN search, reverse arcs from T2 to T1 were strictly prohibited for all variables because it is not logically possible for states at T2 to cause states at T1.

#### Markov Blankets

From this network, we calculated the first- and second-degree Markov blankets for each time point, resulting in all variables capable of fully determining viral suppression. The first-degree Markov blanket of *Viral Suppression* consists of its parents, children, and the parents of its children. The second-degree Markov blanket includes the first-degree Markov blanket of *Viral Suppression* along with the first-degree Markov blankets of all variables within the first-degree Markov blanket of *Viral Suppression*. To assess the predictive power of the data, we calculated the area under the receiver operating curve (AU-ROC) for the first-degree Markov blankets for *Viral Suppression 1* and *Viral Suppression 2*.

#### Combinatory Conditional Probability and Myopic Value of Information Analysis

After identifying the optimal DCBN, we focused on extracting the most meaningful relationships by analyzing combinatorial conditional probabilities of the most relevant variables. We determined which variables should be included in the combinatorial probability calculation as follows: First, we included all variables that were in the second-degree Markov blanket at time points 1 and 2. Then, we excluded those that met any 2 of the 3 criteria: 1) changed only minimally (< 0.01) or not at all with a change in the viral suppression distribution, 2) were among the top 10 variables with the highest normalized Shannon entropy at T1 or T2, and 3) were associated with viral suppression at T1 and T2 with a chi-square P-value > 0.2. In general, we included both time points. However, we only included the T1 variable if its corresponding T2 variable was only connected to T1, meaning its only connection to the rest of the network was through its corresponding T1 variable. This left 18 variables, with the number after the variable indicating if it was a T1 or T2 variable: *Any Drug Use 1, Any Drug Use 2, Born In US, Social Support System 1, Social Support System 2, Emotional Feelings 1, Language, MCM Agency Size 1, MCM Agency Size 2, Neighborhood SES Vulnerability, Provider Rating 1, Provider Rating 2, Race/Ethnicity, Tobacco Use 1, Provider HCR Score 1, Provider HCR Score 2*, and the complementary *Viral Suppression* variable (time point 1 or 2). Using these variables, conditional probabilities for being virally suppressed at T1 and T2 given different combinations of the selected variables were calculated to determine which combination of variable states provided the most information on viral suppression status at T1 and T2. Due to the serial connections in the optimal DCBN structure and the high correlation among *Born In US*, *Neighborhood SES Vulnerability*, *Race/Ethnicity*, and *Language,* we conducted four separate analyses, each including only one of these variables. This approach maintained computational efficiency while preserving essential information, reducing each analysis to 14 variables. We assessed the significance of each configuration by calculating 95% credible intervals. For the maximum probability of being virally suppressed, we considered the configuration significant if the lower bound of the configuration’s credible interval was larger than the upper bound of the credible interval for the marginal probability of being virally suppressed. For the minimum probability of being virally suppressed (i.e., a high probability of not being virally suppressed), we considered the configuration significant if the upper bound of the configuration’s credible interval was lower than the lower bound of the credible interval for the marginal probability of being virally suppressed. We further filtered the significant configurations by removing configurations that contained a subset of another configuration. In other words, we removed configurations with superfluous variables so that only the most parsimonious results were considered. Finally, we removed configurations that contained variables in which all possible variable states appeared in the set of significant configurations, as that implies that the specific variable does not contribute to a change in the probability of being virally suppressed. We applied this procedure to four different possible outcomes: (1) high probability of viral suppression at T1, (2) high probability of viral suppression at T2, (3) low probability of viral suppression at T1, and (4) low probability of viral suppression at T2. Using the network structure, we then used myopic value of information (VOI) and influence scores [77] to examine the paths of influence to viral suppression at T2 for all variables that appeared in the significant configurations of each of the four outcomes described above.

#### Moderation Analysis

Finally, to assess moderation of the time-varying significant psychosocial factors (e.g., *Tobacco Use* and *Any Drug Use*) on viral suppression by the significant health care and provider variables (*Provider Rating, Provider HCR score, and MCM Agency Size*), the probabilities and 95% credible intervals were calculated for changing from no tobacco use to tobacco use and no drug use to drug use, with the scenarios of low, medium and high provider rating and provider HCR score and small vs. large MCM Agency Size. The Florida International University Institutional Review Board approved the study.

## Results

There were 1545 clients who met the analysis inclusion criteria of at least 2 health assessments and viral loads at both time points and being assigned one of the 50 providers for whom we had PCC data; 6 clients had no ZIP Code and were excluded leaving 1539 for analysis. The first time point (T1) for individuals in the group ranged from January 2020 to July 2022; T2 ranged from August 2020 to December 2022. The median time between T1 and T2 was 7 months with a range of 5 to 34 months.

Of the 1539, 1327 (86.2%) were virally suppressed at T1, and 1347 (87.5%) at T2 (Table 1). At T2, 144 (9.4%) were newly suppressed, and 124 (8.1%) were newly non-suppressed (not in Table). The associations between viral suppression and demographic, neighborhood, psychosocial, and health care environment factors are presented in Tables 1 and 2.

**Table 2:** Viral suppression by health care environment and patient centered care factors at time points 1 and 2

Variable	Time point 1							Time point 2						
	Total	Suppressed		Not suppressed				Total	Suppressed		Not suppressed			
	N	n	%	n	%	Test sta-tistic	*P*-value^a^	N	n	%	n	%	Test sta-tistic	*P*-value^a^
Total	1539	1327		212				1539	1347		192			
Health Care Environmental Factors Measured at Time Points 1 and 2														
*MCM agency size*														
≥ 500 clients	1456	1265	95.3	191	90.1	9.81	0.002	1454	1283	95.3	171	89.1	12.32	0.0004
< 500 clients	83	62	4.7	21	9.9			85	64	4.8	21	10.9		
*MCM agency mental health treatment*														
Yes	1212	1042	78.5	170	80.2	0.30	0.59	1206	1056	78.4	150	78.1	0.007	0.93
No	327	285	21.5	42	19.8			333	291	21.6	42	21.9		
*MCM agency substance use treatment*														
Yes	483	418	31.5	65	30.7	0.06	0.81	482	410	30.4	72	37.5	3.90	0.05
No	1056	909	68.5	147	69.3			1057	937	69.6	120	62.5		
*Provider patient volume*														
< 100 RWP patients	178	151	11.4	27	12.7	0.33	0.85	184	160	11.9	24	12.5	0.75	0.70
100–199 RWP patients	505	436	32.9	69	32.6			509	441	32.7	68	35.4		
≥ 200 RWP patients	856	740	55.8	116	54.7			846	746	55.4	100	52.1		
Provider Patient Centered Care Scores Measured at Time Points 1 and 2														
*Provider HCR score*														
Low: < 47.03444 (mean-SD)	294	249	18.8	45	21.2	12.64	0.002	293	250	18.6	43	22.4	5.68	0.06
Medium: 47.03444–50.45315	930	787	59.3	143	67.5			935	813	60.4	122	63.5		
High: ≥ 50.45316 (mean + SD)	315	291	21.9	24	11.3			311	284	21.1	27	14.1		
*Provider knows as person*														
Low: < 0.84032	1285	1109	83.6	176	83.0	0.04	0.84	1286	1123	83.4	163	84.9	0.28	0.59
High: ≥ 0.84032 (mean + SD)	254	218	16.4	36	17.0			253	224	16.6	29	15.1		
*Provider rating*														
Low: < 9.15732 (mean-SD)	307	263	19.8	44	20.8	7.14	0.02	289	259	19.2	30	15.6	0.35	0.35
Medium: 9.15732–9.77111	1152	987	74.4	165	77.8			1170	1016	75.4	154	80.2		
High: ≥ 9.77112 (mean + SD)	80	77	5.8	3	1.4			80	72	5.4	8	4.2		
*Provider asks about challenges*														
Low: < 0.31382 (mean-SD)	305	255	19.2	50	23.6	2.78	0.25	306	270	20.0	36	18.8	1.32	0.52
Medium: 0.31382–0.59763	988	862	65.0	126	59.4			980	851	63.2	129	67.2		
High: ≥ 0.59764 (mean + SD)	246	210	15.8	36	17.0			253	226	16.8	27	14.1		
*Provider asks about worries*														
Low: < 0.40133 (mean-SD)	262	220	16.6	42	19.8	2.89	0.24	269	232	17.2	37	19.3	1.94	0.38
Medium: 0.40133–0.69856	894	768	57.9	126	59.4			889	774	57.5	115	59.9		
High: ≥ 0.69857 (mean + SD)	383	339	25.6	44	20.8			381	341	25.3	40	20.8		
*Provider communication*														
Low: < 14.90164 (mean-SD)	282	244	18.4	38	17.9	2.00	0.37	255	255	18.9	32	16.7	1.18	0.55
Medium: 14.90164–15.57635	1122	972	73.3	150	70.8			1112	967	71.8	145	75.5		
High: ≥ 15.57636 (mean + SD)	135	111	8.4	24	11.3			140	125	9.3	15	7.8		

*MCM* Medical Case Management, *HCR* Health Care Relationship, *RWP* Ryan White Program. Please note that totals of column percentages may slightly exceed 100% due to rounding. a. Chi-square

### Dynamic Causal Bayesian Network Structure

**Fig. 2:**
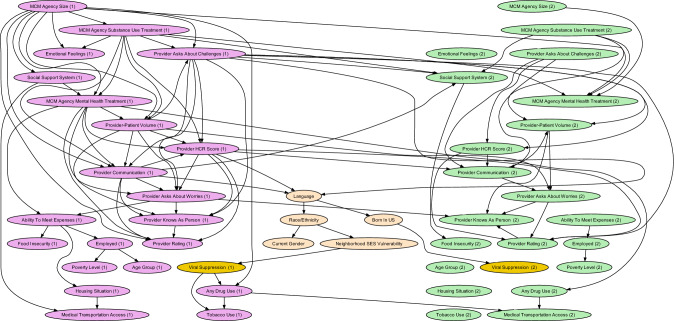
Best Fitting Dynamic Causal Bayesian Network for T1 and T2. (The number in parenthesis after the variable name indicates if it is the variable from T1 or T2. The arcs from T1 to T2 for the same variable are grayed out for ease of clarity. Purple ovals are T1 variables, and light green ovals are T2 variables. Tan ovals are the variables that were only measured at T1, and gold is used for viral suppression). Figure created with Banjo (v2.2.0) and Graphviz (v8.1.0)

Figure 2 reports the best-fitting DCBN structure, which has *Viral Suppression* and 21 other variables at each time point and 5 time-invariant variables. In the figure, the purple ovals are the T1 variables, light green ovals are the T2 variables, tan ovals are the variables that were only measured at T1, and gold is used for viral suppression. The arcs depict statistical relationships between the nodes.

### Markov Blankets

**Fig. 3:**
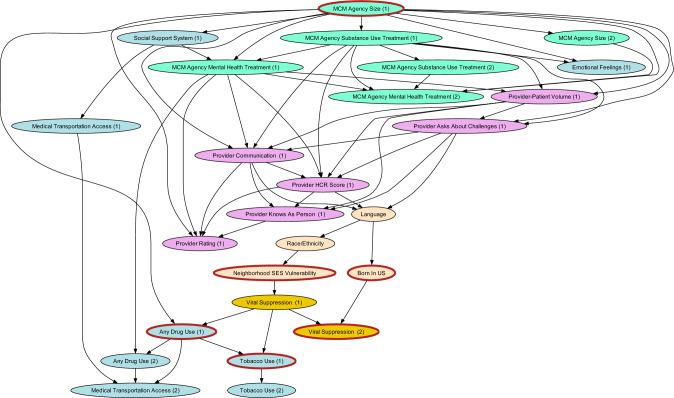
Second-Degree Markov Blanket for T1 from the Dynamic Network. (The number in parenthesis after variable name indicates if it is the variable from T1 or T2. Purple ovals are provider variables, light green ovals are medical case management (MCM) variables, tan ovals are psychosocial and demographic variables, and gold is used for viral suppression. First-degree Markov blanket variables are circled in red). Figure created with Banjo (v2.2.0) and Graphviz (v8.1.0)

**Fig. 4:**
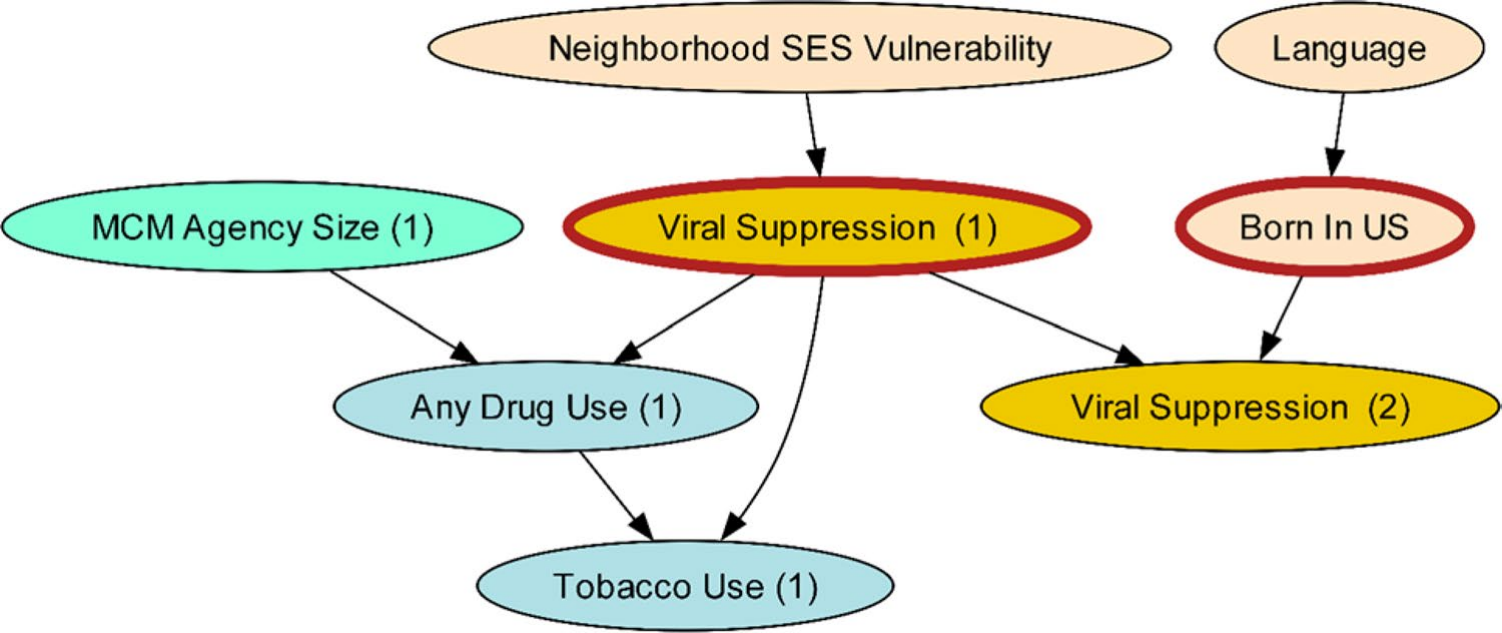
Second-Degree Markov Blanket for T2 from the Dynamic Network. (The number in parenthesis after the variable name indicates if it is the variable from T1 or T2. Light green oval is a medical case management (MCM), tan ovals are psychosocial and demographic variables, and gold is used for viral suppression. First-degree Markov blanket variables are circled in red.). Figure created with Banjo (v2.2.0) and Graphviz (v8.1.0)

The second-degree Markov blanket for *Viral Suppression* at T1 is depicted in Fig. 3 and that for *Viral Suppression* at T2 is depicted in Fig. 4. In both figures, the variables that were in the first-degree Markov blanket are circled in red. The variables in the first-degree Markov blanket for *Viral Suppression 1* included *Born In US*, *Neighborhood SES Vulnerability, Any Drug Use 1*, *Tobacco Use 1, MCM Agency Size 1,* and *Viral Suppression 2*. The first-degree Markov blanket for *Viral Suppression 2* only included *Viral Suppression 1* and *Born in US*. The AU-ROC for the T1 first-degree Markov blanket was 0.674 (confidence limits 0.633 − 0.716), and 0.670 (confidence limits 0.639 − 0.719) for the T2 first-degree Markov blanket, indicating fair predictive performance and suggesting that the model is somewhat effective at distinguishing viral suppression/non-suppression using only the variables in each of the first-degree Markov blankets.

### Myopic Value of Information and Combinatory Conditional Probability Analysis Results

**Fig. 5:**
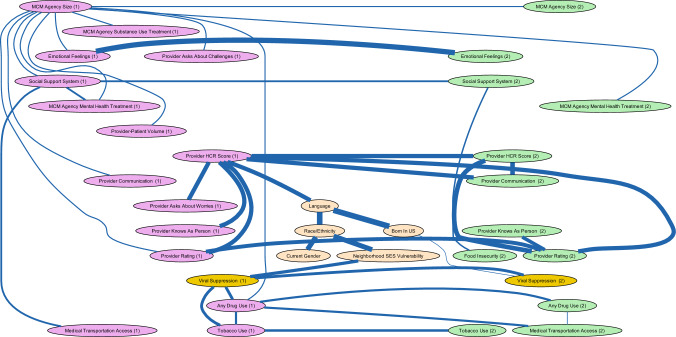
Myopic value of information (VOI): Statistically significant increase in viral suppression. (The number in parenthesis after the variable name indicates if it is the variable from T1 or T2. Larger variable VOIs have thicker paths. Purple ovals are T1 variables, and light green ovals are T2 variables. Tan ovals are the variables that were only measured at T1, and gold is used for viral suppression). Figure created with Graphviz (v8.1.0)

**Fig. 6:**
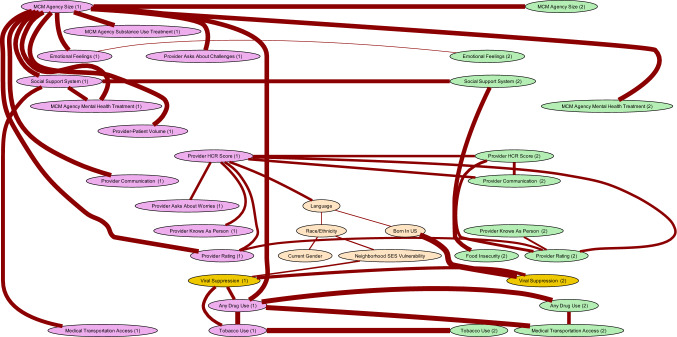
Myopic value of information (VOI): Statistically significant decrease in viral suppression. (The number in parenthesis after the variable name indicates if it is the variable from T1 or T2. Larger variable VOIs have thicker arcs. Purple ovals are T1 variables, and light green ovals are T2 variables. Tan ovals are the variables that were only measured at T1, and gold is used for viral suppression). Figure created with Graphviz (v8.1.0)

Figures 5 and 6 depict the myopic VOI results for a statistically significant increase in viral suppression and a statistically significant decrease in viral suppression respectively. The thickness of each path indicates how crucial that specific piece of information is for forecasting viral suppression (Fig. 5) or non-suppression (Fig. 6) at T2. A key difference in the myopic VOI results is that *Provider HCR Score*, *Provider Rating, Provider Asks About Worries*, and *Provider Knows As Person* are more important for predicting high probability of viral suppression, while *MCM Agency Size*, *MCM Substance Use Treatment*, *Social Support System*, *MCM Agency Mental Health Treatment*, and *Provider-Patient Volume* are more important for predicting low probability of viral suppression. Further, in the Myopic VOI for statistically significant decrease in viral suppression (Fig. 6), *Any Drug Use* and *Tobacco Use* had wide direct paths to *Viral Suppression 1* indicating that these two variables were important in predicting lack of viral suppression.

Based on the combinatory probability analysis and myopic VOI, high provider rating, Hispanic ethnicity, no drug or tobacco use, not US born, Spanish language, and residential neighborhood with high SES predicted the highest probability of viral suppression at T1 (Table 3). *Provider Rating* was only significant when either the ethnicity was Hispanic or the language Spanish or for those foreign born. Living in a relatively wealthy neighborhood (lowest tertile of SES vulnerability) was the only variable that was significant on its own. Those variables predicting the lowest significant probability of viral suppression were using tobacco, using drugs, and attending a small MCM agency (< 500 clients) (Table 4). Being virally suppressed at T1 was the most consistent predictor of a high probability of suppression at T2 followed by being foreign born (Table 5). However, if virally suppressed at T1, a high HCR trust score at either time point, being foreign born, Spanish language, Hispanic ethnicity, large MCM agency, living in a relatively wealthy neighborhood, and not using tobacco or drugs also strongly predicted T2 viral suppression. Independent of *Language*, *US born*, and *Race/Ethnicity*, large MCM agency size, not using tobacco or drugs, and living in a relatively wealthy neighborhood were significant predictors of a high probability of viral suppression at T2. *Provider HCR Score* at T1 and T2 were the only significant predictors for people who were either foreign born or Spanish speaking. Factors predicting the lowest probability of viral suppression at T2 were not being suppressed at T1, low provider rating, born in US, and English language (Table 6). *Provider Rating* was only a significant variable when people were also born in the US.

**Table 3:** Sets of variables with significant high probability of viral suppression at time point 1 (T1)

	Probability of viral suppression	Lower bound, upper bound	States observed						
			Neighborhood SES vulnerability	Provider rating (T1)	Race/ethnicity	Tobacco use (T1)	Language	Any drug use (T1)	Born In US
1	0.911864	0.8862, 0.9321	Least socially vulnerable (most wealthy)						
2	0.881274	0.9413, 0.9996		High^a^	Hispanic	No			
3	0.880899	0.9315, 0.9995		High		No	Spanish		
4	0.879729	0.9448, 0.9996		High	Hispanic			No	
5	0.879421	0.9362, 0.9995		High			Spanish	No	
6	0.879227	0.9413, 0.9996		High		No		No	No

*SES* socioeconomic status. ^a^”High” means greater than 1 standard deviation above mean

**Table 4:** Set of variables with significant low probability of viral suppression at time point 1 (T1)

	Probability of viral suppression	Lower bound, upper bound	States observed		
			Tobacco use (T1)	Any drug use (T1)	Medical case management agency size
1	0.0874825	0.0042, 0.4593	Yes	Yes	Small (< 500 clients)

**Table 5:** Sets of variables with significant high probability of viral suppression at time point 2 (T2)

	Probability of viral suppression	Lower bound, upper bound					States observed						
			Viral load suppression (T1)	Provider HCR score (T1)	Provider HCR score (T2)	Born in US	Language	Race/ ethnicity	MCM agency size	Tobacco use (T1)	Any drug use (T1)	Any drug use (T2)	Neighborhood SES vulnerability
1	0.925962	0.9082, 0.9486	Suppressed		Middle^a^	No							
2	0.925962	0.8925, 0.9564	Suppressed	High^b^		No							
3	0.923578	0.9185, 0.9611	Suppressed		Middle		Spanish						
4	0.923578	0.9123, 0.9569	Suppressed	Middle			Spanish						
5	0.923578	0.8943, 0.9595	Suppressed	High	High		Spanish						
6	0.923578	0.8924, 0.9588	Suppressed				Spanish						
7	0.91747	0.8994, 0.9347	Suppressed					Hispanic					
8	0.905539	0.892, 0.9237	Suppressed						Large (≥ 500 clients)				
9	0.905503	0.8982, 0.9293	Suppressed							No			
10	0.905503	0.8947, 0.9259	Suppressed								No		
11	0.905487	0.8961, 0.9272	Suppressed									No	
12	0.89418	0.8994, 0.9425	Suppressed										Least vulnerable (wealthiest)
13	0.893358	0.8965, 0.9322	Suppressed					Hispanic		No	No		
14	0.893356	0.8957, 0.9317	Suppressed					Hispanic		No		No	

*HCR* Health Care Relationship, *MCM* Medical Case Management, *SES Vulnerability* socioeconomic status social vulnerability index. ^a^ “Medium” is between the mean minus the standard deviation and the mean plus the standard deviation. ^b^ “High” means greater than the mean plus the standard deviation

**Table 6:** Sets of variables with significant low probability of viral suppression at time point 2 (T2)

	Probability of viral suppression	Lower bound, upper bound	States observed				
			Viral load suppression (T1)	Provider rating (T1)	Provider rating (T2)	Born In US	Language
1	0.558824	0.137, 0.7007	Not	Low^a^		Yes	
2	0.558824	0.137, 0.7007	Not		Low	Yes	
3	0.600563	0.4913, 0.704	Not				English

^a^ “Low” is less than the one standard deviation below the mean. The first and second sets are for the same individuals

In sum, high HCR trust scores at T1 and T2 were associated with high probability of viral suppression at T2 among those virally suppressed who were not born in US or Spanish speaking. High provider rating was significantly associated with viral suppression at T1, but only for the subgroup that was either Hispanic, Spanish speaking, or not born in US. On the other hand, low provider rating was associated with low probability of viral suppression at T2 among those not suppressed at T1, but only for those born in the US.

### Moderation Analysis Results

Because *Provider HCR Score*, *Provider Rating*, and *MCM Agency Size* emerged as the most important health care environment variables, these three variables were assessed for their moderating influence on the association of the two most influential modifiable psychosocial variables (*Tobacco Use* and *Any Drug Use*) with viral suppression. However, there was no statistically significant evidence of *Provider HCR Score*, *Provider Rating*, or *MCM Agency Size* moderating the association between viral suppression and tobacco use or drug use.

## Discussion

In this longitudinal analysis, viral suppression was relatively stable with just a slight increase from T1 at 86.2% to T2 at 87.5% for the entire cohort. However, that overall result masks differences among individuals because 9.4% of the group went from non-suppressed to suppressed, and 8.1% went from suppressed to non-suppressed. Thus, 17.5% of the individuals in the group had changes in their viral suppression status between just two time points, highlighting the importance of analyzing viral suppression longitudinally.

The modifiable factors that emerged as most important in predicting viral suppression in the dynamic causal Bayesian analyses were the psychosocial factors of not using illegal drugs and not using tobacco and the health care environment factors of higher provider rating, higher provider Health Care Relationship Trust Score, and large medical case management size. The most important non-modifiable factor was lower neighborhood socioeconomic vulnerability and being foreign born with Hispanic ethnicity and Spanish language being weaker factors.

In multiple steps of the analysis (determining the first- and second-degree Markov blankets, the myopic VOI, and the combinatory probability analysis), both drug and tobacco use emerged as significant predictors of lack of viral suppression. The association of use of illegal drugs with lack of viral suppression has been well established in other studies [7, 13–19] and is likely due to the direct effects of some drugs such as cocaine on viral replication [78], interactions with antiretroviral therapeutic drugs [79], and the mediating factor of nonadherence [17, 79]. Likewise, the association of tobacco use and lack of viral suppression has been firmly established [21–27]. It may be due to nicotine and other tobacco components directly affecting viral replication [80, 81]. It may also at least partly be due to confounding as tobacco use commonly accompanies alcohol use, illegal drug use, and depressive symptoms [21, 82], and each of those factors are associated with lack of adherence and viral suppression [7–10, 12, 32]. We were unable to measure alcohol use because the question in the RWP health assessment referred to alcohol use in the previous year not to current alcohol use. We also had no good measurements for mental health, and it is highly likely that illegal drug use was underreported. Finally, tobacco use may be a coping mechanism for other life stressors that we are unable to measure that interfere with ART adherence and thus viral suppression. Regardless, our results suggest that addressing substance use including tobacco use will be a necessary step to improving rates of viral suppression among people with HIV.

With respect to the provider factors, *Provider HCR Score*, *Provider Rating,* and *Provider Knows As Person* were important for predicting viral suppression based on two of the analyses (second Markov blanket for *Viral Suppression 1* and the myopic value of information analysis). However, the results of the combinatory conditional probability analysis indicated that higher provider HCR score and provider rating predicted viral suppression only among those who were either Hispanic, Spanish speaking or not born in the US, which warrants future investigation. Further, the strength of the influence of *Provider HCR Score* and *Provider Rating* was not as large as that of drug use or tobacco use as evidenced by the lack of provider variables in the first-degree Markov blanket. Finally, we found no evidence that *Provider HCR Score* or *Provider Rating* had a statistically significant moderating effect on the association between tobacco use and drug use with viral suppression. However, this analysis was based on the very small number of people who started tobacco use (n = 20) or drug use (n = 23) from T1 to T2. Thus, we likely did not have the power to detect moderation. Overall, though, our results are consistent with several other studies which have found an association between stronger patient provider relationships and viral suppression in cross-sectional analyses [38, 39, 46, 53], including a cross-sectional analysis of 2019 Ryan White Program data [54]. However, our results are not consistent with two studies among women with HIV [45, 50] and one among men [52], that found no association between measures of patient-provider relationship and viral suppression despite patient-provider relationship measurements being at the participant level.

A large MCM agency size was associated with high probability of viral suppression at T2. To understand this finding, it is important to note that all the large MCM agencies were multi-site federally qualified health centers that provided comprehensive care including specialized care, pharmacy, and mental health services. Thus, the finding may be related to having multiple co-located services or at least the services within one system. A systematic review of studies assessing co-location of services and viral suppression reported one study showing a benefit of co-located mental health services with HIV care, but an inconsistent association for drug abuse treatment with two studies finding a positive association and three showing no association with viral suppression [83]. Similarly, the review reported inconsistent results for co-location of multiple ancillary services and concluded that despite some positive findings, more high-quality studies are required to assess the benefit of service co-location. A recent report of pharmacy co-located services with HIV care including integration of pharmacists in care demonstrated clinically important improvements in viral suppression overall and particularly for those with mental illness and substance use [84]. An additional explanation for the observed benefit of being served by a large MCM agency is that the large MCM agencies tend to serve a demographically diverse population including clients with low to medium SES; whereas, the small MCM agencies tend to serve those with low SES. Although we did have household income as a percentage of Federal Poverty level as a variable in the DCBN, it is possible that there were sociodemographic differences such as educational level that we did not account for.

Residing in a low SES vulnerable (i.e., wealthier) neighborhood was associated with a higher probability of viral suppression, and based on the combinatorial probability analysis results, the effect was not limited to any racial/ethnic group. Neighborhood SES vulnerability was an index based on multiple SES factors including poverty level, household income, unemployment, and education data for the ZIP Code. Surprisingly, while that variable emerged as an important predictor of viral suppression, none of the individual level socioeconomic measures (e.g. *Poverty Level, Food Insecurity)* did. This suggests that some characteristics of neighborhoods that are less socioeconomically vulnerable may be assisting people with HIV in maintaining their health. Candidate characteristics that we did not measure include neighborhood social capital, safety, as well as fewer illegal drug or alcohol venues within the neighborhood. Other research supports the fact that neighborhood SES is associated with viral suppression. A systematic review of studies published through 2022 found that many, although not all, studies have found that living in a neighborhood with more poverty or more unemployment was associated with lack of viral suppression; in contrast, living in a neighborhood with a more educated population was associated with viral suppression [85]. More recent studies found that neighborhood SES was associated with viral suppression in an analysis of people with HIV in the US and Canada [86] and that hot spots of lack of sustained viral suppression were associated with neighborhood levels of social disadvantage [87]. The results of our study and others highlight the importance of better characterizing the mechanisms of how neighborhoods impact health among people with HIV to inform neighborhood-level interventions.

Findings from several analyses (i.e., the first-degree Markov blanket and myopic VOI) indicated that being foreign born strongly predicted viral suppression. Being Hispanic and Spanish-speaking were also statistically significant factors for a high chance of viral suppression at T1, but in only one of the analyses—the combinatory probability analysis—suggesting they are weaker predictors of viral suppression than being foreign-born. That being foreign born was associated with viral suppression is consistent with national findings and other studies. Surveillance data indicate that although foreign-born individuals in the US are disproportionately more likely to be diagnosed late with HIV, they are more likely to be linked to care and be virally suppressed within 6 months of diagnosis [88]. Similarly, an analysis of 2015 − 2017 data from the Medical Monitoring Project found that foreign-born individuals were more likely to be virally suppressed than US-born individuals [89]. While one would predict that foreign-born individuals would be disadvantaged due to language barriers, concern about immigration status, and lack of insurance [47], these barriers likely are less of a concern, at least for Spanish speakers, in the Miami-Dade Ryan White Program, because there are many Spanish-speaking providers and staff, and lack of insurance is a criterion for entry into the program.

## Limitations

A key limitation of this study is that the psychosocial factors including tobacco use and drug use variables were obtained from the administrative data. These data were entered by medical case managers during their health assessments of clients. The questions administered during the health assessment were not validated questions, and the quality of this information likely varies by the skill level and diligence of the specific medical case managers. It is also unknown if medical case managers used the exact wording of questions listed in the needs assessment database as medical case managers communicate with clients in a way that they believe is most effective. It is also highly likely that socially undesirable behaviors such as illegal drug use were underreported. Additionally, we could not examine alcohol use because the question that was available was for alcohol use during the last 12 months, and our two time points were within 12 months in most cases. A further limitation is we were only able to examine two time points due to the study encompassing the COVID-19 Pandemic, when clients were obtaining fewer viral load measurements. We were also unable to examine more time points because the Miami-Dade County RWP migrated in spring 2020 to the Provide Enterprise database system, a system with different variables from those of the previous database system, which prevented us from using data prior to May 2020. It would be important to examine changes over longer periods of time and examine more time points. Additionally, our provider measures were pooled responses from patients of providers taken from a survey. Thus, the measurement is the provider’s overall PCC scores and not how the specific individual in the administrative dataset would necessarily have rated the provider. Another limitation is that the results may not be generalizable to people with HIV who are out of care. The people in the administrative dataset as well as the group of participants surveyed about their provider’s patient-centered care practices were all in the RWP and thus in care at some point. Also, the results may not be generalizable to people with HIV not served by the RWP as RWP clients are more likely to be virally suppressed than people who are not RWP clients [13].

## Conclusion

To our knowledge, this is the first reported study examining longitudinal changes in multiple barriers (e.g. substance use, tobacco use) and enablers (provider patient-centered care) on the probability of viral suppression using DCBN. It is important to replicate this study with data that would allow examination of more time points over a longer period of time and in other geographic areas. Nevertheless, we found that provider PCC factors, tobacco use, and drug use are modifiable factors that are significantly predictive through DCBN analysis. These results highlight the need to improve systems to promote strong patient-provider relationships and address drug and tobacco use with accessible and adequate substance use treatment.

## Supplementary Information

Below is the link to the electronic supplementary material.

Supplementary file1 (DOCX 17 KB)
