# Supplementary material for: Using Dynamic Causal Bayesian Networks to Assess the Role of Patient-Centered Care and Individual-Level Barriers on Viral Suppression Changes Among a Cohort of People with HIV
Source: AIDS Behav. 2026 Feb 22;30(8):2564–83. doi: 10.1007/s10461-026-05082-w (PMC13064917; doi:10.1007/s10461-026-05082-w)
Supplement: Supplementary file 1 [file 10461_2026_5082_MOESM1_ESM.docx]

**Supplementary Table 1** Questions used to obtain summary measures of provider’s patient-centered care practices

**Provider Health Care Relationship (HCR) Score**

*Responses coded as none of the time (0), some or a little of the time (1), occasionally or a moderate amount of time (2), most of the time (3), all of the time (4) were totaled for each survey responded and averaged by provider.*

1. How often does [this provider] discuss options and choices with you about your HIV care before health care decisions are made?
2. [This provider] is committed to providing the best care possible.
3. [This provider] is sincerely interested in me as a person.
4. [This provider] is an excellent listener.
5. [This provider] accepts me for who I am.
6. [This provider] tells me the complete truth about my health-related problems.
7. [This provider] treats me as an individual.
8. [This provider] makes me feel that I am worthy of his/her time and effort.
9. [This provider] takes the time to listen to me during each appointment.
10. I feel comfortable talking to [this provider] about my personal issues.
11. I feel better after seeing [this provider].
12. How often does [this provider] consider your need for privacy?
13. How often do you think about changing to a new health care provider? (reverse coded)

**Provider Rating** (from Consumer Assessment of Health Care Providers and Systems [CAHPS] Survey)

Using any number from 0 to 10, where 0 is the worst provider possible and 10 is the best provider possible, what number would you use to rate [this provider]? *(range from 0 to 10). Ratings averaged by provider.*

**Provider Asks About Challenges** (from CAHPS Survey)

In the last 12 months, did [this provider] or someone from [this provider’s] office ask you if there are things that make it hard for you to take care of your health? *(yes (1), no (2), don’t know). Responses averaged by provider.*

**Provider Asks About Worries** (from CAHPS Survey)

In the last 12 months, did you and [this provider] or someone from [this provider’s] office talk about things in your life that worry you or cause you stress? *(yes (1), no (2), don’t know). Responses averaged by provider.*

**Provider Communication** (items from CAHPS Survey)

Responses coded as never (1), sometimes (2), usually (3), always (4), and don’t know and were totaled for each survey responded and averaged by provider.

1)	In the last 12 months, how often did [this provider] spend enough time with you?

2)	In the last 12 months, how often did [this provider] use medical words you did not understand? (reverse coded)

3)	In the last 12 months, how often did [this provider] talk too fast when talking with you? (reverse coded)

4)	In the last 12 months, how often did [this provider] listen carefully to you?

**Provider Knows As Person**

Does [this provider] know you as a person? *(yes (1), no/don’t know (0)). Reponses averaged by provider*
